# Trigeminal neuralgia: therapeutic strategies to restore quality of life

**DOI:** 10.22514/jofph.2024.024

**Published:** 2024-09-12

**Authors:** Diego Villegas Díaz, Gabriela Guerrero Alvarado, Alan López Medina, José Francisco Gómez Clavel, Alejandro García Muñoz

**Affiliations:** ^1^Aragon Dental Clinic, Faculty of Higher Studies Iztacala, National Autonomous University of Mexico, 057130 Nezahualcóyotl, EM, Mexico; ^2^Research Laboratory in Dental Education, Dental Surgeon Career, FES Iztacala, UNAM, 54090 Tlalnepantla, EM, Mexico; ^3^Almaraz Dental Research Laboratory, Dental Surgeon Career, FES Iztacala, UNAM, 54714 Cuautitlán Izcalli, EM, Mexico

**Keywords:** Trigeminal neuralgia treatment, Facial pain, Diagnosis, Neuropathic pain, Surgical treatment, Neuralgia, Epidemiology, Dental pain

## Abstract

Trigeminal neuralgia (TN) usually affects the maxillary and mandibular branches of the fifth cranial nerve. Although the condition is primarily unilateral, few cases of bilateral manifestation have been reported. TN is uncommon; however, it significantly affects patients’ quality of life because the neuropathic pain worsens over time. Paroxysmal pain is triggered by mechanical stimuli or environmental factors. Diagnosis is usually based on clinical findings, including pain triggered by palpation of distinct areas; nevertheless, imaging studies, such as magnetic resonance imaging, are always used to rule out a secondary cause. TN can be caused by several factors, such as trauma and neurovascular compression, or could be idiopathic, which complicates its treatment. Although several studies focused on TN have been reported, a treatment modality with 100% efficacy is lacking. The first-line treatment is pharmacological; however, surgery may be required if symptoms persist.

## 1. Introduction

Pain is an annoying feeling and emotional experience associated with a potential for tissue damage. It affects 4–13 people per 100,000 inhabitants. Although pain is subjective, the manifestations may help the treating clinician in the diagnosis [1, 2].

The causes of facial pain include tooth pain, myofascial pain, temporomandibular joint disorders, cluster headaches, idiopathic facial pain and trigeminal neuralgia (TN) [1]. TN, which is also known as tic doloreux, is characterized by an episode of unilateral pain in the region supplied by one of the three branches of the trigeminal nerve, which provides afferent innervation to the multiple cavities in the facial skeleton and the dermatomes in this region [3].

The trigeminal nerve has three branches: V1 (ophthalmic, purely sensory), V2 (maxillary, purely sensory) and V3 (mandibular, sensory/motor). The sensory fibers travel to the trigeminal ganglion (also known as the Gasserian ganglion) in Meckel’s cave. The fibers then travel to the spinal trigeminal nucleus, where they synapse with second-order neurons whose fibers travel to the ventral posteromedial nucleus in the thalamus. Finally, the information is transmitted to the sensory cortex. TN is usually unilateral, with a predilection for the right side. Few patients present with bilateral pain, and in approximately 36%–42% of patients, the V2 and V3 branches are affected.

The diagnosis is based on the exclusion of other unrelated pathological processes with similar clinical features, such as neuralgia of the ninth and tenth cranial nerves; oral or facial pain related to organic problems of the concerned organs; altered vision, smell, taste or chewing functions; and pain due to facial skeletal damage.

The purpose of this review was to summarize the information on TN, including its etiology, clinical manifestations, diagnosis and therapeutic options, for health personnel, particularly the general/specialist dentist who plays an important role in the diagnosis, prognosis and treatment. Furthermore, the review aimed to provide an in-depth and updated understanding of TN for informed decision-making to facilitate the maximum improvement in patients’ quality of life.

## 2. Materials and methods

Using the BIDI UNAM Digital Library we had have access to the PubMed, EBSCO, ScienceDirect, Springer, ProQuest, BMJ, Health Library, Elsevier and SciELO database were used for get the articles.

## 3. Results

These results were obtained until 31 May 2024. The searches retrieved a total of 20,121 articles (Table 1), of which 32 articles related to TN were included in the review.

**Table 1.** Keywords searched.

Keywords	Results
Trigeminal neuralgia	10,738
Facial pain and trigeminal neuralgia	2432
Trigeminal neuralgia diagnosis	5509
Dental pain and trigeminal neuralgia	619
Trigeminal neuralgia incidence	768
Laser therapy in trigeminal neuralgia	55
Total	20,121

*The searches retrieved a total of 20,121 articles.*

To select the articles used in this work, the most recent articles with representative information according to year and content were used.

### 3.1 Incidence

The incidence of TN is 4.3 per 100,000 people per year, and the incidence is higher in women than in men (5.9:3.4) [4].

TN is more frequent on the right side than on the left side in patients with multiple sclerosis but not in patients with idiopathic neuralgia. Patients with multiple sclerosis, malignant tumors, trauma, infections and systemic lupus erythematosus are more likely to develop bilateral TN [5].

### 3.2 Diagnosis

The differential diagnosis of TN includes trigeminal autonomic cephalopathy, posttraumatic or postherpetic pain, and other facial pains. The first-line treatment is prophylactic administration of sodium channel blockers, and the second-line treatment is neurosurgical intervention [6].

Determining the location of pain in the dermatomes of the fifth cranial nerve is more important than identifying actions that can trigger pain [7]. Magnetic resonance imaging (MRI) is routinely performed for patients with suspected TN because of the ability to detect neurovascular compression and exclude other diagnoses by visualizing the trigeminal ganglion, nerve and related structures [8].

### 3.3 Etiology

TN is classified into three categories according to the etiology. TN occurring without an apparent cause is called idiopathic. Classical TN occurs due to compression of the trigeminal nerve root by a vessel, whereas TN provoked by a major neurological disease, such as a tumor of the cerebellopontine angle or multiple sclerosis, is classified as secondary TN [9]. Magnetic resonance imaging (MRI) is the preferred method for examining the trigeminal nerve because it reveals changes in the morphology of the trigeminal nerve root and vascular compression in classical TN. However, MRI frequently shows blood vessels in contact with the nerve root in asymptomatic individuals. Therefore, the diagnosis cannot be based on MRI findings alone [9].

The diagnosis of secondary TN is usually based on the identification of a major neurological disease.

Cerebellopontine angle tumors are observed in 15% of patients with secondary TN. Most of these are benign, and they usually compress the root near its entry into the pons. Furthermore, TN can be caused by vascular compression at the entrance of the trigeminal nerve root [10].

According to the neurovascular compression theory, nerve damage due to compression can produce atrophy, hypertrophy or demyelination of the nerve, causing the nerve to misfire, resulting in pain [11]. Furthermore, compression can trigger paroxysmal ectopic discharges [9].

### 3.4 Symptoms

Patients with TN tend to have poor quality of life, and patients with severe TN may attempt suicide owing to uncontrollable pain. TN most frequently affects the second (maxillary) or third (mandibular) division of the trigeminal nerve [3].

Mechanical stimuli that trigger pain commonly include light touch, talking, chewing, tooth brushing or washing the face. Patients usually have more than one trigger area, the most common being the nasolabial area, lips, chin, cheek and alveolar gingiva [12].

Symptoms of TN tend to deteriorate over time with reduced responsiveness to medication despite increased dosage and prescription of additional agents. The definition of treatment success varies between medical and surgical studies on TN. In medical studies, treatment success is typically defined as achieving at least 50% pain relief compared to that at baseline, whereas surgical studies generally consider complete pain relief as treatment success [13].

Individuals experiencing facial pain often find themselves navigating between different medical specialties. Owing to the nature of pain, history and localization, many patients initially seek consultation with dentists. Some patients may have a dental origin for the pain, allowing dentists and maxillofacial surgeons to identify a treatable cause [14].

### 3.5 Treatment

Treatment of TN can be classified as pharmacological, surgical and alternative therapy.

#### 3.5.1 Pharmacological treatment

Currently, pharmacologic management is the treatment of choice, drugs such as carbamazepine, oxcarbazepine, gabapentin and lamotrigine, among others (Table 2). However, 100% pain relief cannot be guaranteed. Therefore, treatment should be selected based on the etiology of TN [15].

**Table 2.** Pharmacological treatment and its dosage.

		Dosages
Anticonvulsivants		
	Carbamazepine	100 mg up to 1200 mg per day
	Oxcarbazepine	900 mg up to 2400 mg per day
	Gabapentin	900 mg up to 3600 mg per day
	Pregabalin	75 mg up to 600 mg per day
	Lamotrigine	100 mg up to 600 mg per day
	Phenytoin	300 mg up to 600 mg per day
Antispasmodic		
	Baclofen	25 mg up to 135 mg per day
Toxin		
	Botulinum toxin	25–75 International Units per infiltration

*(Gambeta et al. [17], 2020), (Latorre et al. [19], 2023), (Boto [20], 2010).*

The International recommendation about surgical treatment is for patients who have resistant pain or who cannot tolerate medications owing to adverse effects [16].

The carbamazepine blocks the voltage-gated sodium channels resulting in inhibition of action potentials, reduction of synaptic transmission and stabilization of the membrane potential in hyperexcitable neurons [17]. This drug seems to be more effective relieving pain, this drug is considered the gold standard for the initial medical treatment of trigeminal neuralgia, but also causes adverse effects such as drowsiness, dizziness, rash, liver damage and ataxia and has the potential for multiple drug interactions [18]. For the same reason that there are adverse effects, other medications are used to mitigate the pain, which are explained in Table 2.

#### 3.5.2 Surgical treatments

##### 3.5.2.1 Rhizotomy

The most commonly used techniques include 3 different techniques: percutaneous balloon compression, percutaneous radiofrequency rhizotomy and percutaneous glycerol rhizotomy [17].

In cases where open surgery is not feasible, percutaneous minimally invasive procedures may be attempted. These procedures are ablative in nature, destroying nerve fibers and, consequently, varying degrees of facial anesthesia.

Radiofrequency rhizotomy uses thermal energy to damage preganglionic trigeminal rootlets, providing immediate pain relief in up to 97% of cases with a 42% recurrence rate at 5 years. The extent of pain relief depends on the depth of facial anesthesia, as expected from an ablative procedure [21].

##### 3.5.2.2 Vascular decompression

Since vascular compression is the most widely accepted cause of primary TN, microvascular decompression may be the most rational treatment option. The initial success rate, mortality, and recurrence rate for this procedure are 92.7%, 0.7% and 2% per year, respectively. Procedural complications, including cerebellar injury, hearing loss, facial paralysis and cerebrospinal fluid fistula, occur at a frequency of <1% and in 15% of cases, no significant vascular compression is observed [10].

In patients with insufficient symptom relief or those who cannot tolerate the treatment, adjunctive drug therapy or monotherapy with medications such as gabapentin, pregabalin, lamotrigine or baclofen may be attempted. Common medications such as over-the-counter analgesics or opioids are usually ineffective. Neurosurgical treatment options consist of either microvascular decompression or percutaneous treatment of the lesion.

Non-destructive surgical treatment: microvascular decompression is the treatment of choice in patients with confirmed contact between the trigeminal nerve and cerebral vessels, which are refractory to pharmacological management. It is a non-ablative microsurgical procedure performed under general anesthesia through a 3-cm craniotomy behind the ipsilateral ear. Under a microscope, the trigeminal nerve is identified and its relationships in the cerebellopontine cistern are explored for compressions, most frequently by the superior cerebellar artery; however, they can also be of venous origin, such as by the superior petrosal vein complex [22]. The offending blood vessels are dissected and either secured by adhering to the tentorium or separated from the nerve using a piece of felt [23].

A previous study on drug-resistant TN reported better pain relief and recurrence rates with microvascular decompression than those with gamma-knife surgery [24].

##### 3.5.2.3 Alternative treatments

Previous studies provide sufficient evidence for acupuncture analgesia [25]. Acupuncture not only inhibits the supraspinal central nervous system region but also induces the combined action of the endocrine and pain-control systems. Furthermore, acupuncture affects the local nerves, reducing their degree of direct pain sensitivity. Regarding the effectiveness of acupuncture for patients with TN, compared with carbamazepine (the gold-standard treatment for TN), acupuncture reduces pain (very low confidence of evidence), response rates (very low confidence of evidence) and the number of adverse events (very low confidence of evidence). However, the evidence is insufficient to recommend its use.

Several studies have reported that compared to placebo, botulinum toxin type A (BTX-A) provides clinically significant benefits in the treatment of TN in terms of the proportion of responders, mean number of paroxysms per day, and visual analog scale scores at the end of follow-up. Furthermore, the effect of BTX-A appears to be relatively long-lasting (at least 3 months) [26].

Other treatments for TN include stereotaxic radiosurgery, percutaneous destructive neurosurgical techniques (chemical neurolysis with glycerol), percutaneous balloon compression, cryotherapy and peripheral block with alcohol. These will not be delved into based on the objectives of this study [27].

#### 3.5.3 Laser therapy

The laser is a painless, easy to apply and non-invasive method that uses light energy generated by atomic excitation that emits photons. The physical process that allows the laser to work is called stimulated emission. Low-level laser therapy (LLLT) has been applied clinically to a wide variety of disorders. This therapeutic method has proven to be effective, less invasive and free of serious side effects for numerous diseases [28].

Multiple studies have shown that low-intensity laser therapy could be considered for the treatment of trigeminal neuralgia. The laser relieves pain without side effects, making it particularly useful for patients with neuralgia who tolerate pharmacological treatment poorly [16].

LLLT has been used in treatment of different diseases especially chronic pains and has been reported as an effective method for alleviating pain [29]. Its pain-relieving mechanism involves the reduction of histamine, bradykinin, acetylcholine and prostaglandin E2, while simultaneously increasing the expression of precursor messenger ribonucleic acid (mRNA) for endorphins, adenosine triphosphate (ATP) and enkephalins [30]. Clinical studies of the effects of LLLT on injured nerves have revealed an increase in nerve function and improved myelin production capacity [16].

Finally, it has been shown in animal models to be effective in promoting axonal growth in damaged nerves [16].

## 4. Discussion

TN is a pathologic process mainly characterized by recurring episodes of intense short duration pain in the area of innervation by the sensory branches of the trigeminal nerve.

This condition, known since ancient times, has been extensively researched; however, several aspects remain unexplored. Pain triggers typically include innocuous mechanical stimuli such as light touch, talking, chewing or washing the face.

Owing to the several potential etiologies of TN, obtaining a definitive diagnosis can be challenging. Primary neuralgia can be easily diagnosed using MRI and must be used to diagnose secondary neuralgia [31].

The optimal approach for diagnosing any of the three types relies on patient history and physical examination because specific laboratory tests to confirm TN are lacking.

TN is classified into three etiological types: idiopathic, classic (caused by vascular nerve compression) and secondary (related to major neurological diseases). Treatment is typically initiated with medications such as anticonvulsants, muscle relaxants and neuroleptic agents. When medications fail to alleviate pain, surgical approaches such as microvascular decompression or ablative percutaneous procedures are considered.

Surgical techniques range from microvascular decompression to radiofrequency ablation, with varying success rates and potential complications such as facial anesthesia. In addition, analgesic blockade using lidocaine, BTX-A and acupuncture can be applied, although their effectiveness and duration of pain relief remain unclear.

Although therapeutic options contribute to treatment success, achieving complete efficacy can be challenging.

However, a cure is not the primary objective, as these treatments initially help manage the disease. Various studies have identified multiple alternatives to expedite progress toward the desired goal. However, comprehensive information is required to decide whether these alternatives offer infallible benefits, pose greater harm due to invasiveness or yield null results. Therefore, the exploration of treatments based on different philosophies or expert viewpoints should continue.

Leclercq *et al*. [23]. reported that the therapeutic approach is primarily “medical”, that is, focused on pharmacological treatment. This involves administering antiepileptic drugs such as carbamazepine, oxcarbazepine, gabapentin or lamotrigine to stabilize hyperexcited neuronal membranes, inhibit repetitive neuronal discharges and reduce synaptic propagation of nerve impulses [23]. However, the same authors suggested that drug-based treatment may fail and proposed implementation of a predominantly surgical therapy. We believe this is an extreme approach, as the authors did not consider the potential outcomes of drug treatment, almost implying a false crown for pharmaceutical interventions.

Rhizolysis involves the application of high-frequency electrical current or radiofrequency energy through an electrode to induce coagulation and denaturation of proteins in the affected tissue. The authors support this technique based on favorable results for several years; however, they acknowledge the risk of losing afferent function in these nerve pathways, resulting in a more damaging, incapacitating, and detrimental treatment than the pathological process itself [18].

In contrast, Wang *et al*. [32] stated full confidence in pharmacological therapy as a promising treatment. They administered carbamazepine and, as an alternative, chose topiramate [32]. Carbamazepine is considered the gold- standard treatment for TN, even by experts such as Ang *et al*. [25] advocating less invasive alternatives like acupuncture.

Carbamazepine reduces painful and uncomfortable symptoms in 100% of treated patients, and in case of adverse effects, it is replaced by oxcarbazepine, with the only drawback being its cost [32]. Wang *et al*. [32] reported that drugs like gabapentin or lamotrigine, recommended by Leclercq *et al*. [23] have an uncertain role in treatment. Maarbjerg *et al*. [6] recommend combined treatment when the maximum doses of carbamazepine and oxcarbazepine cannot be administered due to adverse effects; the commonly used drugs for combination therapy are lamotrigine, baclofen, pregabalin and gabapentin [6].

Wang *et al*. [32] reported that surgical treatments are effective in medication-refractory patients. However, surgery is the last resort, and the authors categorize it as destructive. We believe that in a matter of this magnitude, each opinion should be scrutinized. Although developing new surgical techniques is a significant advancement, information about this pathological process is still in its infancy, and a more in-depth exploration of medication is necessary. In dentistry, the most prominent role in therapy may indeed be pharmacological.

## 5. Conclusions

The diagnosis of TN is often complicated, and several patients go through a trial-and-error phase to find an effective therapeutic approach, as there is no one-size-fits-all solution. Imaging and clinical studies have been used to achieve an accurate diagnosis.

Chronic pain can affect patients’ mood, sleep quality and social relationships. Therefore, emotional support and understanding from family, friends and healthcare professionals is crucial for helping patients cope with the physical and emotional burden of the disease. This is vital to provide effective treatment and improve the quality of life of patients with TN.

Information about the function and effectiveness of alternative treatments for TN is limited, and future studies should investigate these aspects.
